# The association between the use of digital technologies and positive youth development: a systematic review

**DOI:** 10.3389/fpsyg.2025.1552128

**Published:** 2025-07-03

**Authors:** Antonio David Martin-Barrado, Diego Gomez-Baya

**Affiliations:** Department of Social, Developmental and Educational Psychology, Universidad de Huelva, Huelva, Spain

**Keywords:** internet, positive youth development, social media, digital technologies, systematic review

## Abstract

**Introduction:**

Excessive internet use among adolescents has increasingly raised concerns about its potential impact on psychological well-being, including issues such as depression and anxiety. Despite the extensive research on this topic, few studies have examined it through the lens of the Positive Youth Development (PYD) model. This model emphasizes the strengths and skills that enable adolescents to develop into healthy, well-integrated adults. This study aimed to analyze the influence of PYD on children’s and adolescents’ use of the Internet, social media, and Artificial Intelligence.

**Methods:**

A systematic review was conducted following PRISMA guidelines across the Web of Science, Scopus, and PubMed databases from their inception until December 2024. The protocol was registered in PROSPERO (CRD42024602945).

**Results:**

17 quantitative studies (cross-sectional and longitudinal) published between 2012 and 2024 met the inclusion criteria and were evaluated using the Joanna Briggs Institute tool. The results suggest that PYD acts as a protective factor against risk behaviors associated with excessive use of the Internet and social media, such as pornography consumption, sexting, gaming disorders, and cyberbullying. This protective effect is consistent with its influence on other risk behaviors. Furthermore, variables such as emotional self-regulation and family environment were identified as crucial in mitigating these behaviors.

**Discussion:**

The PYD model appears to offer promising strategies for promoting responsible use of digital technologies. However, most studies were conducted in China, suggesting the need for cross-cultural research to support the generalization of the findings. Moreover, research is still needed to address the association between PYD and artificial intelligence use. Finally, the study discusses the implications of these results for future research and practice.

**Systematic review registration:**

https://www.crd.york.ac.uk/PROSPERO/view/CRD42024602945, identifier [CRD42024602945].

## 1 Introduction

In the current digital era, the Internet is fundamental for information retrieval, communication, and entertainment, particularly among adolescents. This stage, marking the transition from childhood to adulthood, is characterized by multiple biological, social, and cognitive changes, alongside increasing autonomy from family. Additionally, sociocultural changes in this century have extended this transition into a stage referred to as emerging adulthood (18–29 years). This phenomenon reflects a delay compared to previous generations in achieving milestones such as securing stable employment, acquiring housing, or starting a family (Arnett, 2013; Arnett and Mitra, 2020).

Some different concepts and uses of the internet have been differentiated in literature, such as internet use, internet addiction, artificial intelligence, cyberbullying or excessive screen time, among others (Bjornsen, 2018). Thus, the complexity of digital technology experiences in youth and their influences on psychological adjustment requires a separate rationale for each topic before reaching an integration (Anderson et al., 2017). Within this context, concerns about Internet addiction have emerged, defined as excessive use of the Internet (Young, 1996), characterized by a constant need for connection and difficulty disengaging from this behavior. This phenomenon is particularly prevalent among individuals under the age of 16, exhibiting symptoms similar to other behavioral addictions (Wartberg et al., 2020). This issue has been recognized within Psychiatry, leading to the inclusion of Internet Gaming Disorder (IGD) in the 5th edition of the Diagnostic and Statistical Manual of Mental Disorders (DSM-5) as a potential diagnosis (American Psychiatric Association, 2013). Subsequently, the World Health Organization (2019) also included it in the 11th edition of the International Classification of Diseases (ICD-11) alongside addictive behavior disorders. Research indicates that factors such as symptoms of depression and anxiety, family issues, deficient social skills, and impulsivity are associated with increased risk of IGD among youth (Fumero et al., 2020; Teng et al., 2021). These findings highlight the importance of developing effective preventive measures within the framework of Information and Communication Technologies (ICT), considering the limited impact of current interventions (King et al., 2018).

Problematic Internet use is influenced significantly by behavioral factors and the reinforcements derived from online activities, rather than solely by the presence of technology itself (Benzi et al., 2023; Grant and Chamberlain, 2014). In this regard, Artificial Intelligence (AI) exacerbates this issue by leveraging user data to generate personalized content and creating a constant sense of reward (Fineberg et al., 2018). However, AI also offers significant benefits for mental health, including tools designed for mood assessment and improvement in patients with depression or suicidal ideation (Joshi and Kanoongo, 2022), and for facilitating the early diagnosis of disorders such as autism spectrum disorder in children (Shahamiri and Thabtah, 2020).

Additionally, there are associated risks such as cyberbullying and sexting. Cyberbullying is an intentional and aggressive behavior carried out through digital means against another person or a vulnerable group (Redmond et al., 2020; Tokunaga, 2010). Other online risks include sexting. This term derived from the words “sex” and “texting,” which involves the transmission of sexual content, sometimes as part of an intimate relationship. However, this behavior poses significant long-term risks due to adolescent vulnerability (Rhyner et al., 2018; Weisskirch and Delevi, 2011; Ybarra and Mitchell, 2014). Gender differences in sexting remain contradictory: some studies report it as more common among women (Ybarra and Mitchell, 2014), others find it more prevalent among men (Garrido-Macias et al., 2021), while some identify no significant differences (Quesada et al., 2018). Furthermore, this behavior can contribute to problematic Internet use (Gomez-García et al., 2020).

Literature has explored these implications during adolescence, identifying adverse effects on cognitive areas, such as memory, analytical thinking, and the interpretation of social cues (Mills, 2016). Schonning et al. (2022) have also linked depression to sleep disturbances, high parental expectations, and social media addiction. A recent meta-analysis highlights that problematic use of these platforms, rather than time spent or intensity of use, serves as the mediating factor explaining the relationship between social media use and depression (Cunningham et al., 2021).

Excessive screen time is associated with diminished psychological well-being, including reduced attention, self-control, curiosity, emotional stability, as well as an increased likelihood of depression and anxiety (Twenge and Campbell, 2018). Furthermore, adolescents with greater Internet addiction tend to exhibit higher impulsivity, more severe depressive symptoms, and less supportive family environments (Marzilli et al., 2020). It is worth noting that the prevalence of excessive Internet use might be underestimated due to factors such as a lack of awareness and the stigma felt by youth when seeking treatment. Moreover, comorbidity with disorders such as depression can obscure the identification of this issue (Fineberg et al., 2018).

Previous research has often operated under a deficit model of adolescence, focusing on risk prevention and portraying youth as passive and problematic agents (Brown and Prinstein, 2011). In contrast to this deficit model, the rise of positive psychology shifted the focus toward adolescent strengths (Lerner et al., 2005a; Lerner et al., 2011). The Relational Developmental Systems Theory underpins the Positive Youth Development (PYD) approach, emphasizing the dynamic, bidirectional interaction between individuals and their contexts. The alignment of ecological assets, such as supportive adults in safe environments, with internal strengths like positive future expectations, facilitates PYD (Lerner et al., 2005b). This approach connects adolescent strengths with family, school, and community resources to optimize and enhance development (Lerner et al., 2011), fostering a healthy, adaptive, and resilient transition into adulthood, irrespective of prior experiences (Geldhof et al., 2014a). This model improved psychological well-being among adolescents and reduced risky behaviors, highlighting the importance of adopting a holistic perspective (Holsen et al., 2017; Waid and Uhrich, 2020).

Within this framework, two models are particularly notable: the 5Cs and Developmental Assets (DA). Regarding the former, Lerner et al. (2005b) identified five interrelated dimensions: (a) Competence, a positive self-perception in areas such as social and academic domains; (b) Confidence, an internal sense of self-esteem encompassing aspects like positive identity and physical appearance; (c) Character, respect for cultural and social norms; (d) Connection, the development of bonds with family, school, and community; and (e) Caring, defined as sympathy and empathy toward others.

The DA model, proposed by Benson et al. (2004), identifies the contextual and personal resources that support PYD. This model comprises 40 assets, divided into 20 Internal Assets and 20 External Assets, each further categorized into four categories. Internal Assets refer to individual characteristics of adolescents, such as Positive Values, Commitment to Learning, Social Competencies, and Positive Identity. External Assets encompass environmental characteristics, including Support, Empowerment, Boundaries-expectations, and Constructive Use of Time.

### 1.1 Justification and objectives

Digital technologies play a critical role during adolescence, shaping social and personal aspects of development. However, research on their relationship with PYD is limited, despite prior studies examining PYD in relation to factors such as substance use, depression, and school satisfaction. Therefore, this systematic review aims to investigate the influence of PYD on how children and adolescents interact with the Internet, social media, and AI. The central hypothesis posits that PYD may play a key role in mitigating the adverse effects of digital technology use.

The findings of this review are expected to provide valuable insights for the design of preventive strategies and the development of guidelines to promote appropriate and responsible use of Information and Communication Technologies (ICT). Additionally, the results may inform interventions aimed at creating a safe digital environment that fosters PYD. This, in turn, facilitates a healthy transition to adulthood in an increasingly digitized world.

## 2 Method

### 2.1 Study design

A systematic review was conducted to examine the influence of PYD on Internet use, social media, and AI, following the PRISMA (Preferred Reporting Items for Systematic Reviews and Meta-Analyses) methodology outlined by Page et al. (2021). This study is registered in the International Prospective Register of Systematic Reviews (PROSPERO) under the identification code CRD42024602945.

Table 1 presents the research question framed using the PICO format.

**Table 1:** PICO format.

Population (P)	Children and adolescents (ages 10–29)
Intervention (I)	Use of Internet, social media and AI
Comparison (C)	Variation in PYD levels/dimensions
Outcome (O)	Studies reporting dimensions of PYD and psychological well-being measures
Research Question	*How does PYD influence the use of Internet, social media and AI among children and adolescents (ages 10–29)?*

### 2.2 Database and search strategy

Electronic searches were conducted on December 26, 2024, in the *Web of Science*, *PubMed*, and *Scopus* databases, with no restrictions on publication date.

Medical Subject Heading (MeSH) terms from the National Library of Medicine were used to develop keywords and optimize the identification of relevant studies. These terms, along with their definitions and synonyms, are detailed in Table 2. Additionally, the following free terms were used: “Positive Youth Development,” PYD, and “Developmental Assets”.

**Table 2:** MeSH terms used in the search.

MeSH term	Meaning	Synonym
Internet	A loose confederation of computer communication networks around the world. The networks that make up the Internet are connected through several backbone networks. The Internet grew out of the US Government ARPAnet project and was designed to facilitate information exchange.	Internet
Social Media	Platforms that provide the ability and tools to create and publish information accessed via the INTERNET. Generally, these platforms have three characteristics with content user generated, high degree of interaction between creator and viewer, and easily integrated with other sites.	Social med* OR mobile social networ*
Artificial Intelligence	Theory and development of COMPUTER SYSTEMS which perform tasks that normally require human intelligence. Such tasks may include speech recognition, learning; visual perception; mathematical computing; reasoning, problem solving, decision-making, and translation of language.	Artificial Intelligence
Adolescent	A person 13 to 18 years of age.	Adolescent* OR teen* OR youth
Child	A person 6 to 12 years of age.	Child*
Student	Individuals enrolled in a school or formal educational program.	Student

The search strategy described in Table 3 outlines the use of Boolean operators *AND* and *OR* across the three databases.

**Table 3:** Search strategy.

Database	Search strategy	Results
Web of Science	internet OR “social med*” OR “artificial intelligence” OR messaging OR “mobile social networ*” (Topic) AND “Positive youth development” OR PYD OR “developmental assets” (Topic) AND youth* OR adolescent* OR teen* OR student OR child* (Topic)	114
PubMed	((internet[Title/Abstract] OR “social med*”[Title/Abstract] OR “artificial intelligence”[Title/Abstract] OR messaging[Title/Abstract] OR “mobile social networ*”[Title/Abstract]) AND (“Positive youth development”[Title/Abstract] OR PYD[Title/Abstract] OR “developmental assets”[Title/Abstract])) AND (youth*[Title/Abstract] OR adolescent*[Title/Abstract] OR teen*[Title/Abstract] OR student[Title/Abstract] OR child*[Title/Abstract])	51
Scopus	(TITLE-ABS-KEY (internet OR “social med*” OR “artificial intelligence” OR messaging OR “mobile social networ*”) AND TITLE-ABS-KEY (“positive youth development” OR PYD OR “developmental assets”) AND TITLE-ABS-KEY (youth* OR adolescent* OR teen* OR student OR child*))	120
Date of search: December 26, 2024		Total: 285

### 2.3 Inclusion and exclusion criteria

The following selection criteria were established: (a) Design criteria, empirical quantitative studies, both cross-sectional and longitudinal, were included to obtain standardized data that enable the identification of significant causal relationships and facilitate comparisons and generalizations; (b) Participant criteria, studies focused on children and adolescents aged 10–29 years; (c) Instrument criteria, studies measuring PYD with a description of the instrument were included; (d) Outcome criteria, articles were required to analyze the relationship between problematic behaviors related to Internet use, social media, or AI, and PYD in their results and discussion sections.

Studies were excluded if they lacked details regarding the data collection period, population description, or demonstrated low scientific quality. No language restrictions were applied.

### 2.4 Data collection and extraction

**Figure 1:**
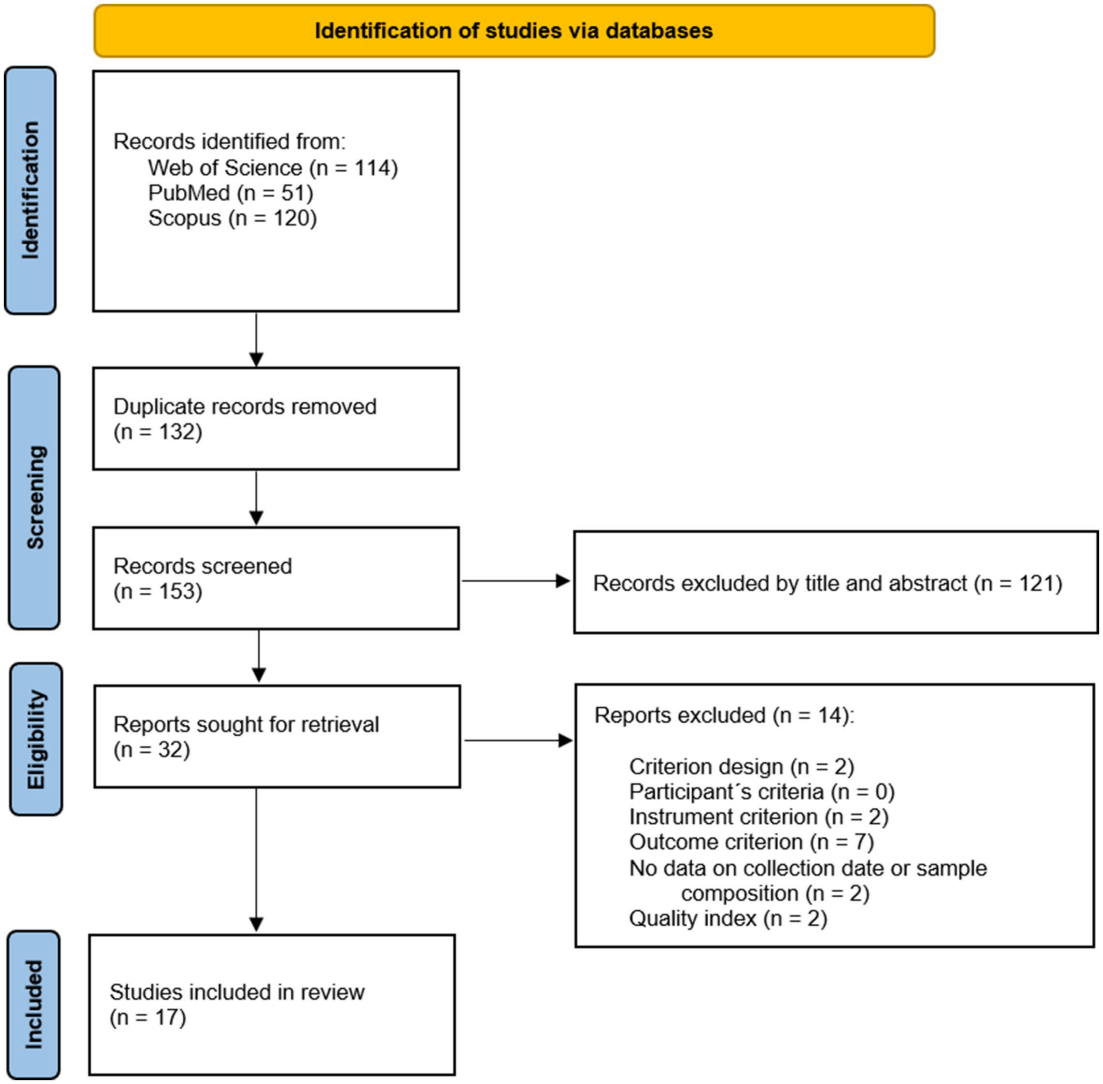
PRISMA flowchart.

The search was conducted independently by two investigators. Each investigator eliminated duplicate studies and selected the studies considered suitable for analysis. Subsequently, both authors reviewed the full text and reached a consensus regarding which studies would be included. The initial search generated a total of 285 potentially relevant results. The screening process was then carried out, and 17 studies were selected for inclusion. The flow diagram followed in the systematic review is shown in Figure 1.

## 3 Results

### 3.1 Characteristics of the studies included

Table 4 summarizes the main characteristics of the studies, all of which were published in English. Of the 17 included studies, 13 were longitudinal and four were cross-sectional. Among the longitudinal studies, seven included two waves, five included three waves, and one included six waves. The total sample comprised 37,015 young students aged 10 to 19 years. Most studies reported no gender bias. However, 12 studies did not report the age range of participants.

**Table 4:** Characteristics of the studies included.

Authors and country	Objective	Methodological designs, and sample characteristics	Date of data collection	Instruments	Main outcomes	JBI
Dou and Shek (2021)* China (Guangdong, Jiangsu, and Jiangxi provinces)	- To investigate the association between PYD and Internet addiction, including the mediating role of life satisfaction.	Longitudinal. *N* = 2,648 students (*M* = 13.12, SD = 0.81; 57.1% men).	2 waves: Starting school year 2016/17–2017/18.	- CPYDS - SWLS - IAT	- PYD attributes were negatively related to Internet addiction. - PYD positively predicted life satisfaction and life satisfaction, in turn, predicted a decrease in Internet addiction. - Life satisfaction was a mediator between PYD and Internet addiction. - Personal strengths, Cognitive-Behavioral competence, and Clear and positive identity were negatively related to Internet addiction. - Prosocial attributes negatively predicted Internet addiction.	11/11
Gan et al. (2023a) China (Hubei province)	- To analyze the mediating and moderating roles of PYD in cyberbullying victimization.	Longitudinal. *N* = 719 students (age 14–18, *M* = 15.95, SD = 0.76; 54.8% men).	3 waves: 2020 (November) – 2021 (May) – 2021 (November).	- CPYDS - CES-D - IGDQ. - E-VS	- IGD and cyberbullying victimization levels were higher in men. - Students in higher grade showed higher levels of PYD and lower levels of depression. - PYD negatively predicted cyberbullying victimization, mediated by IGD. - The association between PYD and IGD was moderated by depression. Higher levels of depression were related to higher IGD.	10/11
Gan et al. (2023b)* China (Hubei province)	- To examine the protective role of PYD against mental health disorders and problematic online behavioral problems during the COVID-19 pandemic.	Longitudinal. *N* = 995 students (*M* = 15.97, SD = 0.77; 54.8% women).	3 waves: 2020 (November) – 2021 (May) – 2021 (November).	- CPYDS - CES-D - E-VS	- PYD attributes negatively predicted depression, cyberbullying victimization, and IGD. - Depression mediated the relationship between PYD and IGD. - Longitudinal evidence indicated that higher PYD levels could predict fewer developmental problems. - Cyberbullying victimization and perpetration mutually mediated their relationship with PYD. - IGD and depression acted as serial mediators in the relationship between PYD and cyberbullying victimization/perpetration.	9/11
Gan et al. (2023c)* China (Hubei province)	- To analyze the relationship between PYD, IGD, and depression.	Longitudinal. *N* = 956 students (*M* = 16.03, SD = 0.75; 54.3% women).	2 waves: 2020 (November) – 2021 (May).	- CPYDS - CES-D - IGDQ	- PYD negatively predicted IGD and depression concurrently and longitudinally. - IGD positively predicted depression concurrently but not longitudinally.	10/11
Gomez-Baya et al. (2022)* Spain (Huelva city)	- To examine the association between PYD and different patterns of Internet use.	Cross-sectional. *N* = 1,038 students (*M* = 14.19, SD = 1.38; 50.1% men).	2020 (autumn).	- PYD-SF - Use and experience of Internet	- Youth who spent less time on the Internet during the week scored higher on PYD. - Young people who experienced negative mood due to not using the Internet or who recognized that they spent excessive time online showed lower PYD. - Using the Internet to search for information or read was positively related to PYD.	8/8
Ma and Shek (2013)* China (Hong Kong)	- To evaluate longitudinal changes in pornography consumption and associated psychosocial factors.	Longitudinal. *N* = 3,325 students (*M* = 12.6, SD = 0.74; 54% men).	3 waves: School year 2009/10–2010/11–2011/12.	- CPYDS - CFAI - Consumption of pornographic materials	- Higher PYD was associated with lower pornography consumption and better family functioning.	10/11
Pistoni et al. (2023) Italy (representative of the country)	- To determine whether the 5Cs and parental control predict different sexting behaviors.	Cross-sectional. *N* = 1866 students (age 13–19, *M* = 16.26, SD = 1.49; 52.52% women).	2018.	- PYD-SF - Sexting behavior - Perceived parental monitoring	- 54% of the sample reported engaging in sexting: 36.2% were passive participants and 17.8% active, with prevalence increasing with age. - Men were more involved in passive sexting. - Men aged 18–19 with high Competence, low Character, and difficulties in self-disclosure to parents were more likely to engage in sexting. Connection was also a significant variable. - Lower Connection was associated with higher participation in active sexting.	8/8
Qin and Gan (2023) China (Hubei province)	- To analyze the association between school assets, self-regulation, self-control, bullying, and IGD.	Longitudinal. *N* = 742 students (age 12–18, *M* = 13.88, SD = 1.99; 53.23% men).	2 waves: Start of the 2021/22 school year – 5 months later.	- School Assets Subscale (DAP) - Intentional Self-regulation - BSCS - OBVQ - IGDQ	- DA negatively predicted IGD. Self-control was a mediating variable in this relationship. - Self-regulation was a moderator in the relationship between school assets and self-control. - Greater school assets may decrease the risk of traditional bullying.	9/11
Shek and Ma (2011)* China (Hong Kong)	- To analyze the prevalence and psychosocial correlates of pornography consumption.	Longitudinal. *N* = 3,638 students (*M* = 13.6, SD = 0.75; 52% men).	2 waves: School year 2009/10–2010/11.	- CPYDS - CFAI - Exposure to pornography - Family history	- Pornography consumption increased longitudinally. This consumption was primarily via the Internet, with men reporting higher levels than women. - Pornography consumption was not associated with socioeconomic status but was linked to immigrant status. - Higher PYD scores were related to better family functioning and lower pornography consumption.	10/11
Shek and Ma (2016)* China (Hong Kong)	- To investigate pornography consumption in relation to sociodemographic and psychosocial variables.	Longitudinal. *N* = 3,291 students (*M* = 12.6, SD = 0.74%; 52% men).	6 waves: School year 2009/10–2015/16.	- CPYDS - CFAI - Consumption of traditional and online pornography	- Online pornography consumption was higher than traditional pornography. - Men showed higher pornography consumption. - Worse family integrity was associated with higher pornography consumption. There was no relationship with economic level. - PYD was shown to be a protective element against pornography consumption. However, Confidence predicted negative outcomes.	10/11
Wang et al. (2023)* China (Chengdu city)	- To examine the relationship between PYD and Internet addiction in youth who experienced COVID-19 lockdowns.	Longitudinal. *N* = 7,985 students (*M* = 10.60, SD = 2.18; 51.65% men).	2 waves: 2019 (December) – 2020 (June).	- CPYDS - IAT	- PYD levels decreased while Internet addiction increased following the COVID-19 lockdown. - Higher PYD predicted lower Internet addiction, particularly in the components of Cognitive-Behavioral competence, Positive identity, and general PYD qualities.	10/11
Xiang et al. (2022a) China (Sichuan, Hubei, and Shaanxi provinces)	- To evaluate the association between cyberbullying and IGD, including the protective role of PYD.	Cross-sectional. *N* = 463 students (age 11–18; *M* = 15.06, SD = 1.48; 57.88% men).	2020 (November) – 2021 (January).	- CPYDS - E-BVS - IGDQ	- PYD was negatively related to both IGD and cyberbullying. Furthermore, PYD mediated the relationship between these variables. - Youth who experienced IGD reported higher rates of participation in online bullying behaviors.	8/8
Xiang et al. (2022b)* China (Two schools in the South)	- To examine the relationship between DA, self-control, and IGD.	Longitudinal. *N* = 1,023 students (*M* = 13.16, SD = 0.86; 49.36% men).	2 waves: 2020 (October) – 2021 (April).	- DAP - CDAI - BSCS - IGDQ	- DA were negative predictors of IGD, influencing it indirectly through self-control.	10/11
Xiang et al. (2022c)* China (Hubei province)	- To analyze the cumulative effects of DA on IGD during the COVID-19 pandemic.	Longitudinal. *N* = 1,023 students (*M* = 13.16, SD = 0,86; 49.36% men).	2 waves: 2020 (October) – 2021 (April).	- DAP - IGDQ	- Higher DA were related to lower IGD concurrently and longitudinally. - Youth showed more Internal than External Assets. Positive Identity was the only asset that showed gender differences, in favor of men. - Internal Assets mediated the relationship between External Assets and IGD longitudinally.	10/11
Yu and Shek (2013)* China (Hong Kong)	- To investigate the prevalence and psychosocial correlates of Internet addiction.	Longitudinal. *N* = 4,106 students in wave 3 (*M* = 14.65, SD = 0.80; 53.7% men).	3 waves: School year 2009/10–2010/11–2011/2.	- CPYDS - CFAI - IAT - ASC	- In the third year, the percentage of Internet addicts decreased. - Men showed higher rates of Internet addictive behaviors, while women reported receiving more parental control. - Family functioning and PYD were negative predictors of Internet addiction.	11/11
Yu and Shek (2021) China (Hong Kong)	- To evaluate the protective role of PYD and positive parenting practices against social media addiction.	Cross-sectional. *N* = 1896 students (age 10–16; *M* = 13.19, SD = 0,52; 52% men).	2019 (February–June).	- CPYDS - BSMAS - CPBS	- PYD acted as a mediating variable between parental responsiveness and social media addiction. Almost 20% of the sample reported spending more than four hours on these platforms, and a similar proportion exhibited significant social media addiction. - The dimensions of Emotional-Behavioral competence, Beliefs in the future, and Spirituality were negatively related to social media addiction. However, Social competence, and Clear and positive identity were positively related.	8/8
Zhang et al. (2024)* China (Shenzhen city)	- To examine the relationships between depressive symptoms and Internet addiction, including individual, family, and school-related predictors.	Longitudinal. *N* = 1,301 students (*M* = 12.46, SD = 0.63; 51.2% men).	3 waves: Between October and November 2016–2017 – 2018.	- CPYDS - CES-D - IAT	- PYD, as an Internal Assets, was identified as a predictor of youth mental health and as a protective factor against Internet addiction and depressive symptoms. The family, as an External Assets, was also shown to be protective. Both variables showed long-term effects. - Other predictive variables were gender and academic performance. - Men showed more Internet addictive behaviors.	11/11

*Does not report age range; ASC, Academic and School Competence Scale; BSCS, Brief Self-Control Scale; BSMAS, Bergen Social Media Addiction Scale; CDAI, Cumulative Development Assets Index; CES-D, Center for Epidemiologic Studies-Depression; CFAI, The Chinese Family Assessment Instrument; CPBS, Chinese Parenting Behavioral Scale; CPYDS, Chinese Positive Youth Development Scale; DAP, Development Assets Profile; E-VS, E-Victimisation Scale; IAT, Internet Addiction Test; IGDQ, Internet Gaming Disorder Questionnaire; OBVQ, The Olweus Bully/Victim Questionnaire; PYD-SF, Positive Youth Development-Short Form; SWLS, Satisfaction With Life Scale.

**Figure 2:**
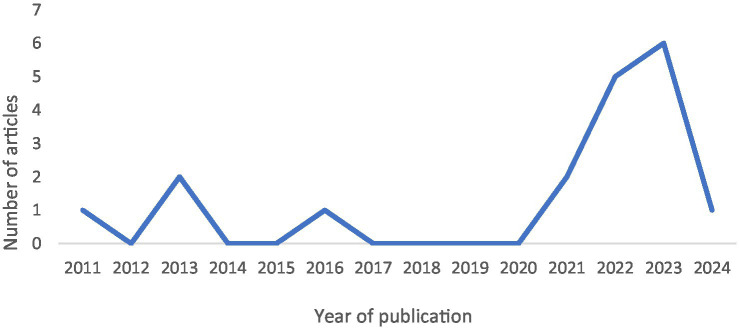
Distribution of articles by year of publication.

Most articles were published between 2021 and 2024, reflecting a marked increase in research activity since 2011. This trend is illustrated in Figure 2.

The journal that published the highest number of articles was *Frontiers in Public Health* (*n* = 5), followed by the *Journal of Pediatric and Adolescent Gynecology* (*n* = 3). Other journals in smaller proportions were: *Current Psychology* (*n* = 2), *Frontiers in Pediatrics* (*n* = 2), *Frontiers in Psychology* (*n* = 1), *International Journal of Environmental Research and Public Health* (*n* = 1), *Journal of Adolescence* (*n* = 1), *PloS ONE* (*n* = 1) and *The Scientific World Journal* (*n* = 1).

**Figure 3:**
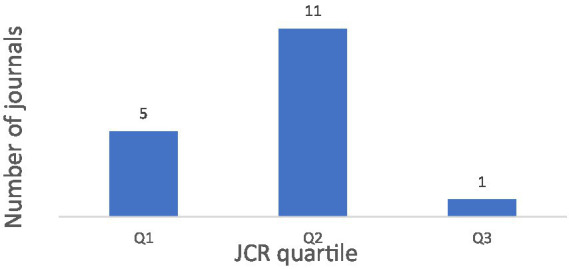
Quartile distribution of the JCR impact factor of included journals.

The Journal Citation Report (JCR) tool was used to classify the journals’ impact factors into quartiles. This distribution is depicted in Figure 3. In 2022, *Frontiers in Public Health* had the highest impact factor (5.1) and was classified in Q1. Conversely, the *Journal of Pediatric and Adolescent Gynecology* recorded the lowest impact factor, with a score of 1.58 in Q3 in 2016. For *Current Psychology* and *Frontiers in Public Health*, 2023 indices were used due to the unavailability of 2024 data. None of the journals belonged to Q4 (Figure 3).

**Figure 4:**
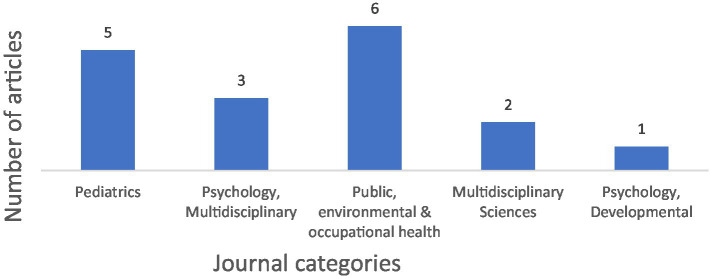
Distribution of journal categories.

Based on the JCR tool, the majority of categories were classified under *Public, Environmental & Occupational Health* (*n* = 6) and *Pediatrics* (*n* = 5) (Figure 4).

There was a predominance of studies carried out with Asian samples (*n* = 15), particularly in China. Only two studies were carried out in Europe: Spain (*n* = 1) and Italy (*n* = 1). No studies included international samples. With the exception of Pistoni et al. (2023), which analyzed a representative sample of Italy, all studies focused on specific areas (e.g., city, province, or region).

### 3.2 Instrument analysis

All studies included sociodemographic variables, and three instruments were utilized to measure PYD. The most frequently used instrument was the *Chinese Positive Youth Development Scale* (CPYDS) developed by Shek et al. (2007), which was applied in 44 (Yu and Shek, 2021), 80 (e.g., Dou and Shek, 2021), and 90-items versions (e.g., Gan et al., 2023a, 2023b, 2023c). These items (e.g., *“My belief is that even though tomorrow will become worse, I will still live in a good manner”*) were divided into 15 subscales: Bonding, Resilience, Social competence, Recognition for positive behavior, Emotional competence, Cognitive competence, Behavioral competence, Moral competence, Self-determination, Self-efficacy, Clear and positive identity, Beliefs in the future, Prosocial involvement, Prosocial norms, and Spirituality. Yu and Shek (2013) used a shorter version of the CPYDS employing four dimensions (Shek and Ma, 2010): Cognitive-Behavioral competencies, Prosocial attributes, Positive identity, and General PYD qualities. The CPYDS utilized a 6-point Likert scale (1 = *Strongly disagree* to 6 = *Strongly agree*) and demonstrated high internal consistency (Cronbach’s *α* ≥ 0.90) across various studies (e.g., Gan et al., 2023a; Zhang et al., 2024).

Xiang et al. (2022b) utilized the *Developmental Assets Profile* (DAP) developed by the Search Institute (2005). This instrument, consisting of 58 items (e.g., *“I have a school that cares about children and encourages them”*), is divided into eight subscales with demonstrated cross-cultural applicability (Scales, 2011). In contrast, Qin and Gan (2023) employed only the school assets subscale of the DAP. This 10-item subscale examines educational factors such as safety and engagement (e.g., “*I am trying to learn new things*”). Both studies used a 4-point Likert scale (1 = *Rarely* to 4 = *Almost always*) and achieved Cronbach’s alpha values close to 0.90.

The *Positive Youth Development – Short Form* (PYD-SF) developed by Geldhof et al. (2014b) was employed in its 32 (Pistoni et al., 2023) and 34-item version (Gomez-Baya et al., 2022). This instrument uses a 5-point Likert scale (1 = *Not true for me* to 5 = *Very true for me*) to assess the 5Cs: Competence, Character, Confidence, Connection, and Caring. For example, items include: *“I am popular among my peers”* (Competence) and *“I think I am good-looking”* (Confidence). The instrument demonstrated reliable Cronbach’s alpha values ranging from 0.64 to 0.87.

Other instruments used to assess variables associated with PYD included the Chinese version of the *Internet Gaming Disorder Questionnaire* (IGDQ), developed by Yu et al. (2017). This questionnaire consists of 11 items scored on a 3-point Likert scale (1 = *Never* to 3 = *Often*). Additionally, the Chinese version of the *Internet Addiction Test* (IAT) by Young and De Abreu (2010) was employed to measure excessive Internet use over the past year using 10 dichotomous items (0 = *No* and 1 = *Yes*). Furthermore, the *Center for Epidemiologic Studies Depression Scale* (CES-D) by Radloff (1977) was applied to assess depression in Chinese adolescents. All these instruments demonstrated strong reliability.

### 3.3 Content analysis

Table 4 summarizes the included articles, providing information about the authors and their countries of origin, the objective of each study, methodological designs, and sample characteristics, the date of data collection, instruments used, main findings, and quality assessments.

A negative relationship was found in six studies between higher PYD scores and lower levels of excessive Internet use (Dou and Shek, 2021; Gomez-Baya et al., 2022; Wang et al., 2023; Yu and Shek, 2013; Zhang et al., 2024) and social media use (Yu and Shek, 2021). Dimensions of the CPYDS (Shek et al., 2007), such as Cognitive-Behavioral competence, Prosocial norms, and Positive identity, were associated with lower excessive Internet use (Dou and Shek, 2021; Wang et al., 2023). Yu and Shek (2021) identified a negative relationship between social media addiction and dimensions such as Emotional-Behavioral competence, Beliefs in the future, and Spirituality. Social competence and Clear and positive identity were positively correlated with addiction. Additionally, PYD mediated the relationship between parental responsiveness and social media addiction. Across two studies, almost 20% of the sample exhibited strong addiction tendencies, with males showing a higher prevalence (Yu and Shek, 2013; Zhang et al., 2024).

Gomez-Baya et al. (2022) demonstrated that youth with higher PYD scores spent less time online during weekdays. In contrast, those who felt unpleasant emotions when offline or who engaged in excessive Internet use reported lower PYD scores. However, online activities such as information seeking and reading were linked to higher scores.

Three variables influenced Internet addiction: PYD, family support, and the adverse effects of COVID-19 lockdowns. PYD served as a mediating and protective factor through life satisfaction (Dou and Shek, 2021). Family support, as an External Asset, showed a protective role against Internet addiction (Xiang et al., 2022b; Yu and Shek, 2013) and depression (Zhang et al., 2024). However, the adverse effects of COVID-19 lockdowns increased addictive behaviors and negatively impacted PYD (Wang et al., 2023).

Four studies demonstrated that higher PYD scores predicted lower pornography consumption and better family functioning (Ma and Shek, 2013; Pistoni et al., 2023; Shek and Ma, 2011, 2016), although Confidence predicted negative outcomes (Shek and Ma, 2016). There was a preference for online formats over traditional ones, and higher pornography consumption was observed among males. Immigrant status influenced consumption patterns, but socioeconomic status did not (Shek and Ma, 2011, 2016). Additionally, Pistoni et al. (2023) found that Connection reduced sexting behaviors among Italian youth. Conversely, high levels of Competence and low levels of Character increased these behaviors. Males engaged more in passive sexting (receiving sexual content), but active sexting (sending sexual content) showed no gender differences. Open communication with parents reduced sexting, but parental control had no significant relation to it.

Seven studies found a negative association between PYD and IGD in both cross-sectional and longitudinal analyses (Gan et al., 2023a, 2023b, 2023c; Qin and Gan, 2023; Xiang et al., 2022a, 2022b, 2022c). DA negatively predicted IGD through self-control (Qin and Gan, 2023; Xiang et al., 2022b, 2022c), with higher self-regulation associated with greater self-control among youth with similar school assets scores. The Internal Asset mediated the relationship between External Assets and IGD, and Positive Identity showed gender differences, favoring males (Xiang et al., 2022c). One study associated IGD with depression concurrently, but not longitudinally (Gan et al., 2023c). Depression mediated the relationship between PYD and IGD, with higher depressive symptoms linked to greater IGD (Gan et al., 2023a, 2023b). Males scored higher in IGD, while students in higher grades showed greater PYD scores and lower depression levels (Gan et al., 2023a).

Finally, four studies examined the influence of PYD on cyberbullying (Gan et al., 2023a, 2023b; Xiang et al., 2022a) and traditional bullying (Qin and Gan, 2023). PYD acted as a protective factor against both cyberbullying victimization and perpetration, with males participating more frequently (Gan et al., 2023a). Additionally, youth with IGD symptoms exhibited higher rates of involvement in bullying behaviors. PYD mediated the relationship between IGD and cyberbullying (Xiang et al., 2022a), while another study identified IGD as the mediating variable (Gan et al., 2023a).

No study was found addressing the associations between the use of AI or the attitudes towards AI and PYD.

### 3.4 Methodological quality assessment

The Joanna Briggs Institute (JBI) tool, developed by the University of Adelaide, was used to evaluate the methodological quality of the included articles. The JBI framework assesses whether a study has minimized or eliminated potential biases in its design or analysis. Two versions of the tool were employed: one for cross-sectional studies (8 items; Moola et al., 2020) and another for cohort studies in longitudinal designs (11 items; Moola et al., 2020). These details are presented in Tables 5, 6. The cut-off points for inclusion were set by consensus of both researchers at 6 and 8, respectively.

**Table 5:** Scores of cross-sectional studies.

Study	JBI	The participants and the environment are described in detail	Clearly defined inclusion criteria	Exposure was validly and reliably measured	Objective criteria to measure the condition	Confounding factors were identified	Strategies for dealing with confounding factors	Valid and reliably measured results	Appropriate statistical analysis was used
Gomez-Baya et al. (2022)	8/8	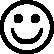	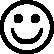	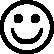	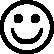	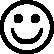	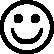	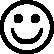	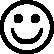
Pistoni et al. (2023)	8/8	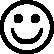	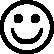	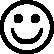	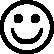	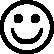	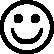	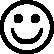	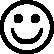
Xiang et al. (2022a)	8/8	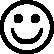	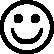	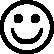	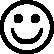	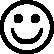	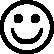	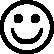	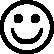
Yu and Shek (2021)	8/8	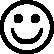	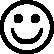	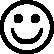	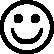	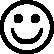	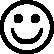	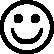	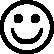

Yes: 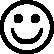, No: 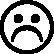, Unclear or Not Applicable: 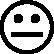.

**Table 6:** Scores of longitudinal or cohort studies.

Study	JBI	Were the two groups similar and recruited from the same population?	Was exposure measured in a similar way to allocate people to both exposure and non-exposure groups?	Was exposure measured in a valid and reliable way?	Were confounding factors identified?	Were strategies to address confounding factors indicated?	Were the participants /groups unaware of the results at the start of the study (or at the time of exposure)?	Were the results measured in a valid and reliable way	Was the follow-up time reported and was it sufficient to produce results?	Was follow-up completed and, if not, were the reasons for lack of follow-up described and explored?	Were strategies to address incomplete monitoring used?	Was an appropriate statistical analysis used?
Dou and Shek (2021)	11/11	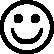	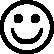	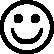	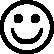	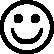	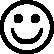	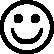	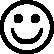	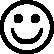	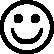	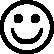
Gan et al. (2023a)	10/11	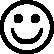	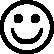	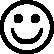	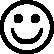	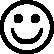	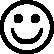	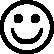	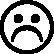	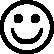	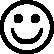	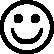
Gan et al. (2023b)	9/11	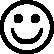	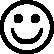	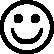	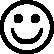	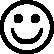	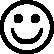	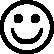	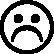	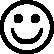	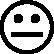	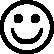
Gan et al. (2023c)	10/11	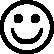	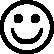	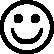	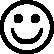	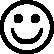	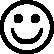	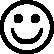	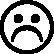	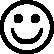	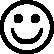	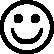
Ma and Shek (2013)	10/11	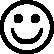	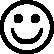	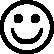	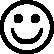	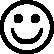	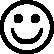	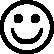	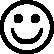	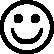	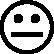	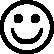
Qin and Gan (2023)	9/11	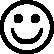	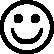	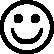	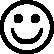	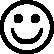	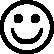	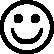	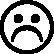	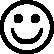	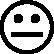	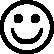
Shek and Ma (2011)	10/11	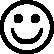	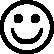	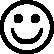	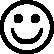	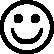	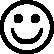	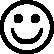	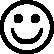	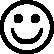	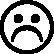	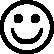
Shek and Ma (2016)	10/11	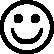	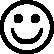	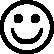	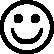	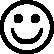	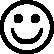	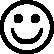	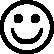	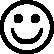	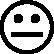	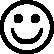
Wang et al. (2023)	10/11	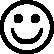	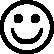	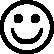	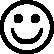	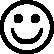	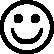	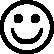	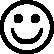	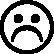	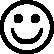	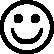
Xiang et al. (2022b)	10/11	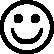	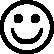	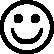	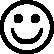	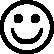	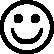	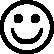	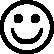	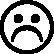	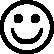	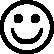
Xiang et al. (2022c)	10/11	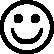	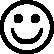	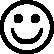	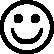	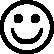	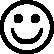	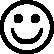	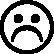	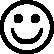	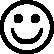	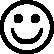
Yu and Shek (2013)	11/11	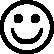	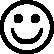	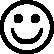	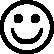	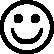	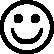	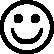	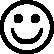	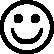	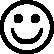	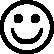
Zhang et al. (2024)	11/11	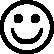	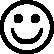	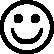	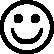	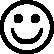	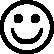	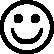	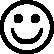	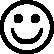	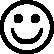	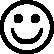

Yes: 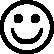, No: 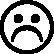, Unclear or Not Applicable: 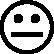.

Seven articles demonstrated strong overall scores, while the remaining studies achieved moderate scores. None of the articles were rated as weak. Furthermore, all studies excelled in identifying and managing confounding factors, employing robust strategies to address these variables.

## 4 Discussion

PYD focuses on enhancing adolescents’ strengths to facilitate a healthy transition into adulthood and reduce risky behaviors, such as substance use (Lerner et al., 2011; Geldhof et al., 2014a; Geldhof et al., 2014b). However, there is a knowledge gap regarding the connection between this framework and the use of ICT. This systematic review aimed to explore whether PYD influences the use of the Internet, social media, and AI. To the authors’ knowledge, this review represents one of the first attempts to systematically address this topic.

This study analyzed 17 articles from an initial pool of 285 studies from Web of Science, PubMed, and Scopus up to December 2024. The bibliometric analysis revealed that these articles fall within the domains of health sciences and social sciences. Although no temporal filters were applied to maximize the number of included studies, the majority were published after 2021, reflecting a growing interest in the topic. Most samples originated from Asia, likely reflecting heightened concerns about the pandemic’s impact on youth mental health and online behavior (Li et al., 2021). In Europe, while data also indicate concerning trends, there is a notable lack of preventive programs (Lopez-Fernandez and Kuss, 2020). No study was observed addressing the relationship between AI and PYD.

The included studies provided strong evidence that higher PYD scores acted as a protective factor against several online risk behaviors, including Internet and social media addiction, pornography consumption, sexting, IGD, and cyberbullying. Since its emergence in the early 21st century, the PYD framework has been extensively studied for its effectiveness in mitigating risk behaviors and promoting healthy transitions to adulthood (Holsen et al., 2017; Lerner et al., 2011; Waid and Uhrich, 2020). These findings support the theory that the benefits of this approach, traditionally applied to risk behaviors such as substance use or delinquency, are equally relevant in the context of ICT.

Certain dimensions of the CPYDS, such as cognitive-behavioral competencies were associated with reduced Internet addiction (Dou and Shek, 2021; Wang et al., 2023). Emotional competence, behavioral competence, beliefs in the future and spirituality were negatively associated with social networking addiction, while social competence and positive identity were linked to increased social media addiction (Yu and Shek, 2021). This paradoxical association may be explained because online communication could provide a space for adolescents to obtain social support and explore peer relationships, what may offer the opportunity to practice their social skills before applying them face-to-face. Moreover, internet usage may help to establish supportive peer relationships, what is also important for adolescents’ healthy identity development. Within the 5Cs framework, the Connection component was associated with reduced sexting behaviors (Pistoni et al., 2023), whereas Confidence and Competence were associated with increased pornography consumption (Shek and Ma, 2016). For instance, excessive self-confidence may lead adolescents to perceive themselves as invulnerable, making them more likely to engage in risky behaviors (Brendgen et al., 2004). Despite Confidence refers to self-esteem and positive self-concept, and Competence is associated with self-efficacy in different life domains, very high scores in these dimensions may show some overstatement and bias in self-perception, as well as invulnerability sense, what may generate some risks (Gomez-Baya et al., 2023). Thus, more self-awareness and critical thinking should be fostered in adolescence to prevent biased self-perceptions and promote better decision-making regarding some risk behaviors (Murphy et al., 2014; Halpern-Felsher and Cauffman, 2001).

Additionally, heightened social influence may increase peer pressure (Allen, 2024). It is important to foster balanced development across PYD scores to prevent unrealistic self-appraisal of personal abilities. External Assets, such as family support, play a fundamental role in fostering PYD (Yu and Shek, 2013; Zhang et al., 2024). Support from adults in an adolescent’s environment promotes the development of a Positive Identity (Jankowska-Tvedten and Wiium, 2023), and a warm, cohesive family climate enhances PYD (Buehler, 2020). Conversely, dysfunctional family contexts elevate the risk of Internet addiction (Aponte-Rueda et al., 2017). These findings emphasize the importance of creating a supportive and nurturing environment to optimize adolescent development and mitigate the risks associated with excessive use of ICT.

Research indicated that men engaged more frequently in behaviors related to Internet addiction (Yu and Shek, 2013; Zhang et al., 2024), sexting (Pistoni et al., 2023), pornography consumption (Shek and Ma, 2011, 2016), and cyberbullying (Gan et al., 2023a). These findings align with prior literature indicating a higher prevalence of externalizing behaviors among males (Garrido-Macias et al., 2021), such as aggressive conduct and behavioral addictions. In contrast, women often experience greater parental control due to societal gender norms and expectations (Yu and Shek, 2013). While increased parental supervision could benefit adolescents of both genders, it may also lead to limited access and increased monitoring of digital activities for women. This differential treatment can potentially result in unequal opportunities for digital engagement and autonomy.

Internet addiction tends to decrease with age. This may be because younger individuals gradually develop greater self-control over time, and the novelty of early access to ICT diminishes (Qin and Gan, 2023; Xiang et al., 2022b, 2022c). Maturity and PYD play a significant role in reducing this addiction (Gan et al., 2023a; Yu and Shek, 2013, 2021). This distinguishes it from other behavioral addictions, which tend to increase over time (Hayes et al., 2020). Further studies are needed to explore this relationship in more depth.

Problematic Internet use can negatively impact various areas of development, including physical activity, academic performance, social relationships, and family communication. These negative consequences can stem from deficits in personal skill development, including self-efficacy, emotional self-regulation, and positive social values, key capacities within the PYD framework (Lerner et al., 2011). One study indicated that young individuals with higher self-regulation showed improved self-control (Qin and Gan, 2023). This finding aligns with a longitudinal study that revealed positive associations between the 5Cs and self-regulation skills. In contrast, those who experienced difficulties in emotional regulation exhibited depressive symptoms and greater involvement in risky behaviors (Gestsdóttir and Lerner, 2007). Moreover, fostering best practices for ICT use could mitigate its adverse effects. Gomez-Baya et al. (2022) highlighted that constructive Internet use, guided by activities such as reading and information-seeking, enhances PYD. This approach supports self-regulation, knowledge acquisition, and critical thinking, which helps build a strong sense of identity and fosters positive social relationships (Lerner et al., 2011).

### 4.1 Limitations and strengths

Several limitations must be considered when interpreting the results. First, most of the samples were drawn from various regions of China, which makes it challenging to generalize the findings to other contexts, such as Europe. Recent reviews have identified similarities in the benefits of PYD between Europe and the United States (Martin-Barrado and Gomez-Baya, 2024), suggesting that further studies in European settings would be beneficial. This overrepresentation of studies with Chinese samples limits generalizability of the results, because of the differential cultural characteristics. As argued Paska and Yan (2011) some differences in internet use and addiction with Western countries can be reported. These authors indicated that internet addiction was more common among students in China than among students in the United States, who have been exposed to and have used the Internet longer than have their Chinese counterparts. The heavy use of internet is perceived as positive by interviewed Chinese students, because it enhances students’ self-identification, closer relationships with friends and bonding with the world, while research results underline that heavy internet use is associated with poor grades, sleep deprivation, and greater risk of emotional problems. In this line, Jackson and Wang (2013) showed that loneliness was a positive predictor of internet use in Chinese samples, while high conscientiousness was a negative predictor of internet use to meet new people. These authors argued that Chinese collectivist culture encourages shared activities, over solitary ones, and values family and friends (i.e., interacting with and caring for them), over the individual self. Thus, cross-cultural studies about PYD, internet use and their different mechanisms and moderators are still needed.

Second, the inclusion of samples collected during the COVID-19 lockdown may have influenced PYD levels. Third, non-peer-reviewed studies, qualitative research, and low-quality studies were excluded. While this exclusion maintains high standards, it may have overlooked relevant studies, particularly in regions with less stringent standards for review or research quality. Future reviews should consider exploring the motivations and concerns of young people through qualitative research. Qualitative designs can provide some insights into youth motivation and meaning making in digital contexts. Fourth, the adolescents in the studies were drawn from school settings, and their ages ranged from 10 to 19 years. It is recommended that future research employs random, representative, and gender-balanced samples. Fifth, all measures were self-reported, incorporating external informants is suggested. These methodological improvements would enhance understanding of the protective mechanisms of PYD in the context of ICT use.

The works included in the present review examined various areas of digital technologies. However, none addressed the influence of AI use within the PYD framework. This gap in the literature presents opportunities for future researchers to investigate whether AI technology can impact youth well-being, as this group represents a substantial audience. Understanding whether PYD can promote appropriate and ethical use of tools such as AI is critical.

Despite these limitations, this systematic review has several strengths. First, the main strength of this review lies in the inclusion of mostly longitudinal studies, allowing for the evaluation of causal relationships and developmental patterns. Nevertheless, additional research in other contexts is needed to enhance the generalizability of the results. Second, the absence of temporal and language restrictions broadened the article pool. Third, the included articles were published in high-impact journals, reinforcing the relevance of the findings. Fourth, the most commonly used instrument for measuring PYD in China was the CPYDS (Shek et al., 2007), with various item versions that could have influenced the results. This instrument has demonstrated excellent reliability and validity in the Chinese population (Shek et al., 2021).

### 4.2 Policy and practical implications

Public policies should actively involve adolescents to ensure their voices are incorporated, as they are the best informants about their online activities. This participatory approach would allow for the design of more effective prevention and intervention programs tailored to their actual needs. It is also recommended to expand information channels for adolescents to prevent the Internet from being their sole primary source of information. Families and schools play a critical role in fostering responsible and appropriate ICT use, as these contexts significantly influence PYD (Jankowska-Tvedten and Wiium, 2023). An effective strategy is to implement psychoeducational programs for parents on safe Internet usage (Fineberg et al., 2018). These programs equip parents to act as guides and allies to their children, promoting positive digital habits. Such interventions are low-cost, quick to implement, and family-centered, creating a safe and supportive environment that reinforces adolescents’ psychosocial and emotional competencies. Several studies in this review emphasized that fostering communication within parent–child relationships reduced adolescents’ engagement in risky online behaviors (Pistoni et al., 2023; Yu and Shek, 2013, 2021). Moreover, this approach aligns with the Relational Developmental Systems Theory, which highlights the importance of family relationships (Lerner et al., 2005a).

To promote youth well-being in the digital age, practical interventions should prioritize socialization within supportive environments such as families and schools, thereby mitigating social isolation. Addressing excessive internet use, particularly among males, necessitates a comprehensive approach that strengthens social and familial relationships. This approach should also foster responsible attitudes towards online risks, thus preventing internalizing behaviors such as anxiety and depression (Buehler, 2020). Furthermore, the implementation of mindfulness techniques and workshops on the responsible use of electronic devices is recommended, as well as prioritizing the quality of digital activities over screen time. Both families and schools must educate youth on responsible online risk management, rather than simple avoidance (Allen, 2024). To prevent cyberbullying, the development of socio-emotional skills, including empathy, self-regulation, and conflict resolution, is crucial, recognizing that PYD and emotional intelligence are interdependent constructs requiring time and appropriate contexts to develop (Schoeps et al., 2018). Finally, schools should also attend to students’ IGD status, integrate PYD attributes into their programs, and implement psychoeducational programs focused on the prevention and intervention of online risks. An example of such a program is Safety.net (Ortega-Barón et al., 2024), which has demonstrated effectiveness among Spanish adolescents in promoting general internet use competencies and preventing online risks such as problematic internet use, IGD, and nomophobia.

### 4.3 Conclusion

This review examined the influence of the PYD model on excessive ICT use, including the use of the Internet, social media, and AI. The available literature indicated that higher PYD scores were linked to less problematic technology use. This result suggests that developing certain assets and dimensions such as self-regulation and family support may be key factors. However, despite these positive findings, research on this topic remains limited. Most studies related to PYD have focused on externalizing risk behaviors, such as substance use, without addressing contemporary challenges such as Internet addiction. As Internet usage continues to grow, PYD-based interventions could be valuable in mitigating the adverse effects of technology misuse, particularly those designed to improve family communication and social skills. In addition, more research across cultures and contexts is essential to develop more effective interventions and to explore emerging issues such as AI. Although these findings are promising, further investigation is needed to support the protective role of PYD in an increasingly digitalized world.

## Data Availability

The original contributions presented in the study are included in the article/supplementary material, further inquiries can be directed to the corresponding author/s.
